# Therapeutic subtypes of knee osteoarthritis: differential treatment effects among predicted endotypes in past clinical trials

**DOI:** 10.1186/s13075-026-03825-7

**Published:** 2026-05-07

**Authors:** Monica T. Hannani, Peder Frederiksen, Morten A. Karsdal, Cecilie L. Bager, Asger R. Bihlet, Karin Tunblad, Fredrik Öberg, Jamie E. Collins, Virginia B. Kraus, David J. Hunter, Jaume Bacardit, Anne-Christine Bay-Jensen, Christian S. Thudium

**Affiliations:** 1 Nordic Bioscience A/S, Herlev Hovedgade 205-207, Herlev, 2730 Denmark; 2 Department of Biomedical Sciences, Faculty of Health and Medical Sciences, University of Copenhagen, Copenhagen, Denmark; 3 NBCD A/S, Soeborg, Denmark; 4 Medivir AB, Huddinge, Sweden; 5 Department of Orthopedic Surgery, Brigham and Women’s Hospital, Boston, MA USA; 6 Harvard Medical School, Boston, MA USA; 7 Duke Molecular Physiology Institute, Department of Medicine, Duke University School of Medicine, Durham, NC USA; 8 Sydney Musculoskeletal Health, Kolling Institute, Faculty of Medicine and Health, The University of Sydney, Arabanoo Precinct, Sydney, Australia; 9 Rheumatology Department, Royal North Shore Hospital, St Leonards, Australia; 10 Interdisciplinary Computing and Complex BioSystems (ICOS) research group, School of Computing, Newcastle University, Newcastle upon Tyne, UK

**Keywords:** Biomarker, Endotype, Endotyping, Enrichment, Osteoarthritis, Subtype, Subtyping, Stratification, Theratype

## Abstract

**Background:**

Molecular endotyping may facilitate the successful development of personalized treatments of knee osteoarthritis (KOA). The aim of this exploratory and hypothesis-generating study was to develop a clinically actionable tool for predicting molecular endotypes of KOA using blood-based biomarkers, and to explore the potential for differential treatment effects across biomarker-based endotypes in prior phase II-III KOA drug trials.

**Methods:**

Fourteen biomarkers from 226 KOA participants from IMI-APPROACH were assessed for a multinomial logistic regression model to predict structural damage, inflammation, and low tissue turnover endotypes. An optimized panel of six serum biomarkers (C2M, C3M, N-MID, PRO-C2, PRO-C4, sCTX-I) was identified quantitatively by testing all biomarker combinations in models adjusting for age, sex, and BMI. These biomarkers were used for endotype predictions in KOA participants from the randomized placebo-controlled trials MIV-711 (*n* = 244) (NCT02705625), salmon calcitonin (*n* = 947) (NCT00486434), and UBX0101 (*n* = 175) (NCT04129944).

**Results:**

The structural damage endotype showed the greatest numerical 26-week reduction in NRS knee pain when treated with MIV-711 (-6.46%; 95% CI: -16.23%, 3.31%). Notably, only the structural damage endotype had a significant two-year reduction in WOMAC pain when treated with salmon calcitonin (-6.19%; 95% CI: -10.55%, -1.83%). Considering the subset treated with salmon calcitonin with the top 20% highest probability of belonging to the structural damage endotype, a 9–15% reduction in standard deviation of the two-year change in WOMAC pain was observed. Enriching the trial for this subset with lower outcome variability could have led to an 18–28% reduction in the needed sample size.

**Conclusions:**

This exploratory study suggests the feasibility of predicting KOA endotypes using a minimal panel of tissue-turnover biomarkers. The observed treatment effects of anti-bone resorptive treatments and reduced outcome variability in the structural damage endotype may subtly indicate that aligning treatments with endotypes and drug modes of action can possibly enhance their therapeutic efficacy. Further investigation is needed to establish the true clinical utility of biomarker-based endotyping. Endotype-informed trial design and recruitment may represent a promising strategy to increase the likelihood of success in future KOA clinical trials.

**Supplementary Information:**

The online version contains supplementary material available at 10.1186/s13075-026-03825-7.

## Background

Osteoarthritis (OA) affects 595 million people worldwide and is one of the most rapidly increasing health conditions globally [1]. However, individuals living with OA remain without clinically approved disease-modifying treatment options as all previous interventional OA trials have failed to meet their primary endpoints. A contributing factor to the plethora of failed OA clinical trials is the failure to account for the substantial heterogeneity within the OA population [2]. One way to address this heterogeneity is to identify molecular endotypes defined by specific disease-driving molecular pathways [3]. Endotyping holds the potential to maximize the likelihood of achieving meaningful therapeutic outcomes in OA. This may facilitate the targeted enrollment of endotype subgroups driven by molecular pathways that biologically match the mode of action of a drug. Current methods for endotyping in OA have relied on diverse analytical techniques with varying degrees of complexity including metabolomics [4, 5], single-cell transcriptomics [6], multi-omics [7], and clustering of biochemical markers [8]. By *k*-means clustering of tissue-turnover biomarkers in 295 knee OA (KOA) participants, the Innovative Medicines Initiative Applied Public-Private Research enabling Osteoarthritis Clinical Headway (IMI-APPROACH) consortium discovered three endotypes driven by structural damage to bone and cartilage, connective tissue inflammation, and low tissue turnover, which were externally recaptured in the Osteoarthritis Initiative (OAI) study [8]. We have recently shown that 54% of the KOA participants of IMI-APPROACH had a longitudinally stable endotype across three visits (spanning approximately 18 months), which is an important aspect for the clinical applicability of molecular endotyping [9]. However, these endotypes have not yet been validated by demonstrating differential treatment effects in clinical trials, a necessary step to establish them as true treatable endotypes, or theratypes [2]. While current methods for endotyping, such as population-based clustering and omics techniques, are instrumental for the understanding of the molecular underpinnings of OA, their applicability in a clinical trial context is limited by their associated costs and complexity.

The use of endotyping as a predictive enrichment tool for clinical trial recruitment has not yet been implemented. A clinically applicable method to successfully and accurately endotype OA participants for future drug trial recruitment is urgently needed to facilitate the development of targeted OA therapies. The enrichment of endotype subgroups that biologically match the drug mode of action in clinical trials can potentially facilitate more effective drug development by increasing treatment effect size and reducing outcome variability, and thereby decreasing the number of participants needed to demonstrate treatment effect. Biomarker-driven clinical trials have become a reality for several diseases, such as oncology [10] and cystic fibrosis [11], and biomarkers may be useful for the identification of endotype subgroups to target for personalized OA drug development [12]. As such, the three biomarker-derived endotypes originally described by the IMI-APPROACH consortium may be suitable for proof-of-concept of clinically applicable biomarker-based endotyping. However, the number of biomarkers used for molecular endotyping influences the applicability and costs in a clinical trial context. Therefore, it is important to develop endotyping tools based on a minimal set of parameters to ensure their feasibility.

This exploratory, hypothesis-generating study aimed to develop a clinically applicable model to predict three molecular endotypes—structural damage, inflammation, and low tissue turnover [8]—using a minimal panel of blood-based biomarkers of tissue turnover and to explore the potential for endotype-specific treatment effects in OA trial data for the first time. To our knowledge, endotyping has not previously been used in a clinical trial context in OA, and we therefore investigated historical OA trial data. As previous OA drug trials with proven effect and available data are very limited, we considered well-known and well-run past OA drug trials with drug modes-of-action hypothesized to align with the pathobiological drivers of the three endotypes. The endotyping concept was applied to three former phase II-III OA drug trials evaluating the efficacy of salmon calcitonin, cathepsin K-inhibiting MIV-711, and senolytic UBX0101 (ClinicalTrials.gov IDs: NCT00486434, NCT02705625, NCT04129944) [13–16]. However, development and approval of all three treatments were halted as these did not meet their primary endpoints. This study explored the potential for differential treatment effects between predicted endotypes and investigated whether they exhibited stronger treatment effects than the entire trial population in historical OA trial data.

## Methods

The aim of this exploratory study was to (i) develop a predictive model to classify participants into one of the three established OA endotypes using a minimal and targeted set of tissue-turnover biomarkers, (ii) apply the prediction model to three prior interventional trials to assign endotypes, and (iii) evaluate the potential differences in treatment effects among the predicted endotype subgroups (Supplementary Fig. 1 and Supplementary Materials). All study participants fulfilled the American College of Rheumatology criteria for tibiofemoral KOA.

### Development of endotype prediction model

#### Model population

To develop a molecular endotype prediction model, longitudinal endotype assignments of KOA participants from IMI-APPROACH were utilized [9]. IMI-APPROACH was a two-year, European, observational cohort of 297 adults with KOA (ClinicalTrials.gov ID: NCT03883568). The inclusion criteria for IMI-APPROACH were primary KOA diagnosis, age ≥ 18 years and a high probability of structural and/or pain progression within two years [17]. The participants had previously been assigned an endotype for each visit (months 6, 12, and 24) through *k*-means clustering of 19 biomarkers, as described by Hannani et al. [9]. The inflammatory endotype was characterized by higher biomarker levels of systemic (high-sensitivity C-reactive protein [hsCRP]) and connective tissue (CRPM and C3M) inflammation, and had the highest proportion of pain progressors over two years [8]. The structural damage endotype was defined by higher biomarker levels of bone (sCTX-I, u-αCTX-I, and N-terminal middle fragment of osteocalcin [N-MID]) and cartilage (uCTX-II) turnover and had the highest proportion of structural progressors over two years. The low tissue turnover endotype was characterized by lower levels of most biomarkers and had the highest relative proportion of non-progressors over two years [8]. Longitudinal data of participants with data available for more than five biomarkers at all visits were included in this study (*n* = 226). Of these, 123 participants showed longitudinally stability of their endotype (assigned to the same endotype at all three visits independently) of structural damage (*n* = 51), inflammation (*n* = 40), or low tissue turnover (*n* = 32) [9].

#### Biochemical marker data and preprocessing

All blood-based and commercially available biomarkers were considered from the IMI-APPROACH (*n* = 14/19) for use in a biomarker-based endotype classification model. The biomarkers were previously measured in an International Organization for Standardization-certified laboratory at Nordic Bioscience A/S and reflected bone formation and degradation (sCTX-I, N-MID, and PRO-C1), cartilage formation and degradation (C2M, C10C, cartilage oligomeric matrix protein [COMP], hyaluronic acid [HA], and PRO-C2), and systemic and connective tissue inflammation (C1M, C3M, CRPM, hsCRP, PRO-C4, and citrullinated vimentin [VICM]) [3, 9]. Biomarker values were natural-log transformed. For each visit, outliers were defined as > Q3 + 1.5 × interquartile range (IQR) or < Q1–1.5 × IQR and were winsorized to the 98th and 2nd percentiles, respectively. Missing data due to insufficient sample volume were imputed using a random forest model with the R package *missForest* (ntree = 300) (Supplementary Table 1) [18].

#### Multinomial logistic regression model for endotype predictions

A multinomial logistic regression model was used to predict molecular endotypes using blood-based biomarkers, age, sex, and body mass index (BMI). To balance the accuracy and applicability of the model, based on preliminary testing of a range of biomarker panel sizes (results not included), the predictions were based on a minimal set of six biomarkers. The input biomarker and clinical data were z-score scaled. A total of 75% of all participants were included in the model training. To increase the reliability of endotype predictions, the model was tested in 25% of the participants with stable endotype assignments across all visits.

To quantitatively determine the optimal set of six biomarkers for endotype predictions, all combinations of six out of all blood-based and commercially available biomarkers were trained (*n* = 3003 unique models), and each model was performed 30 times with resampling. Multinomial ridge regression models were constructed using the *glmnet* R package. The biomarker panel with the highest average sensitivity across all endotypes in the test set was used for the final model.

With the highest average sensitivity of 90% in the test set, the final input parameters for the model were C2M, C3M, N-MID, PRO-C2, PRO-C4, sCTX-I, age, sex, and BMI (Supplementary Table 2).

### Assessment of differential treatment effects among predicted endotypes in prior clinical trial data

#### Trial populations

Three former interventional phase II-III KOA trials were considered for the assessment of endotype-specific treatment effects on primary endpoints. As historical OA drug trials with proven effect and available data that are endotype-targeted are very limited, we included three past OA drugs with drugs mode-of-action that we hypothesized were well-aligned with the pathobiological drivers of the three endotypes.

The oral salmon calcitonin trial (CSMC021C2301) was a two-year, phase III, randomized, multicenter, double-blinded, placebo-controlled trial testing the efficacy of oral salmon calcitonin (Novartis and Nordic Bioscience A/S) in 1176 KOA participants (ClinicalTrials.gov ID: NCT00486434) [13]. Salmon calcitonin acts as an anti-bone resorptive peptide by binding and activating the calcitonin receptor on osteoclasts. The inclusion criteria for CSMC021C2301 were symptomatic KOA diagnosis, age 51–80 years, joint space width (JSW) ≥ 2.0 mm, Kellgren-Lawrence (KL) grade 2–3, Western Ontario and McMaster Universities Arthritis (WOMAC) pain > 150/500 mm, and/or WOMAC function > 510/1700 mm (higher WOMAC scores indicated worse symptoms) in the target knee. The study was randomized 1:1 to 0.8 mg oral salmon calcitonin twice daily or a matching placebo. The primary study outcomes were changes in JSW and WOMAC pain from baseline to month 24 [13]. Longitudinal data were included from participants who completed the study and had measurements for more than five biomarkers at both baseline and month 24, resulting in 435 participants in the placebo arm and 512 in the treatment arm. The biomarkers ARG, C1M, C2M, C3M, CRPM, N-MID, PRO-C2, sCTX-I, uCTX-I, uCTX-II, and VICM had previously been measured [9] while PRO-C4 was quantified in serum for the purpose of this study by Nordic Bioscience A/S.

MIV-711-201 was a 26-week, phase IIa, randomized, multicenter, double-blinded, placebo-controlled trial testing the efficacy of MIV-711 (Medivir AB) in 244 KOA participants (ClinicalTrials.gov ID: NCT02705625) [15, 16]. MIV-711 is a selective cathepsin K inhibitor that reduces osteoclast-mediated bone resorption [14]. Inclusion criteria were primary symptomatic and radiographic KOA diagnosis, age 40–80 years, average Numeric Rating Scale (NRS) knee pain of ≥ 4 and < 10 (0–10 NRS scale; with higher scores indicating worse symptoms) within one week prior to the first visit. The study was randomized 1:1:1 to once-daily oral administration of MIV-711 (100, 200 mg) or matched placebo. The primary endpoint was the change in the average NRS knee pain from baseline to week 26. Longitudinal data of participants who completed the study at baseline and week 26 were included (*n* = 215). The biomarkers C2M, C3M, N-MID, and sCTX-I had previously been measured [14], while PRO-C2 and PRO-C4 were measured in serum in this study.

UBX0101-MUS-201 was a 12-week, phase II, randomized, multicenter, double-blinded, placebo-controlled trial testing the efficacy of UBX0101 (UNITY Biotechnology) in 183 KOA participants (ClinicalTrials.gov ID: NCT04129944) [16]. UBX0101 is a mouse double minute-2 (MDM2)/p53 inhibitor with senolytic properties [16]. Inclusion criteria were KOA diagnosis, age 40–85 years, KL grade 1–4, average NRS daily pain of ≥ 4 and ≤ 9 (0–10 NRS scale; higher scores indicated worse symptoms) for at least five out of seven days during screening. The study was randomized 1:1:1:1 to single-dose intra-articular administration of UBX0101 (0.5, 2.0, or 4.0 mg) or matched placebo. The primary endpoint was the change in WOMAC pain (higher scores indicated worse symptoms) from baseline to week 12. Longitudinal data of participants who completed the study for baseline and week 12 were included (*n* = 145). The biomarkers C2M, C3M, N-MID, PRO-C2, PRO-C4, and sCTX-I were measured in serum in this study.

#### Biomarker-based endotype predictions

The six-biomarker panel (C2M, C3M, N-MID, PRO-C2, PRO-C4, and sCTX-I) was measured in serum at baseline in all three trials. For each trial, the biomarkers were preprocessed as described above. Each participant was assigned an endotype using the multinomial logistic regression prediction model with biomarkers, age, sex, and BMI as input parameters.

### Statistical analysis

All analyses were performed using R version 4.4.2. For each study, least-squares (LS)-mean differences in primary endpoints between placebo and treatment within each endotype were estimated using linear regression models with an interaction term between the treatment and endotype. Age, sex, BMI, KL grade, and baseline values were included as the covariates. LS-mean differences (treatment contrasts) were estimated using the *emmeans* package and were marginalized over sex and KL grade. Differential treatment effects across endotypes were tested using F-tests, comparing the full interaction models to equivalent models without the interaction term between treatment and endotype. Based on the modes of action of the treatments, the structural damage endotype was hypothesized to show the greatest treatment effect of the anti-resorptive agents, salmon calcitonin and MIV-711, whereas the inflammatory endotype was hypothesized to exhibit the strongest treatment effect of the senolytic agent UBX0101. A nominal significance level of 5% was applied. P-values were adjusted for multiple tests using Holm’s method. For MIV-711-201 and UBX0101-MUS-201, the active dose groups were pooled and compared as one to placebo due to sample-size constraints.

The relationship between outcome variability of WOMAC pain and the probability of belonging to each endotype was explored in the treatment arm of CSMC021C2301 (*n* = 512). The standard deviation (SD) of the two-year change in WOMAC pain was computed for every decile cutoff of the endotype membership probabilities obtained from the prediction model. The percent differences between the SD of the two-year change in WOMAC pain of the full population and each endotype probability decile group were calculated. The sample size needed in each treatment arm to demonstrate a statistically significant difference of 32/500 mm in WOMAC pain was calculated with *power.t.test* in R using a power of 96% (as specified in the original study) [13] and the SD of each endotype probability decile group.

## Results

### Biomarker-based endotype prediction model

For the development of an endotype prediction model, longitudinal data from 226 KOA participants from IMI-APPROACH were considered for model training (Table 1) [17]. The participants had previously been assigned to the endotypes (i) structural damage, (ii) inflammation, and (iii) low tissue turnover in a study by Hannani et al. (2025) by *k*-means clustering of 19 tissue-turnover biomarkers [9]. To balance the feasibility and accuracy of the multinomial logistic regression model for use in a clinical trial context, it was decided to base the model on a minimal set of six blood-based biomarkers along with the clinical parameters age, sex, and BMI. The optimal set of biomarkers was determined quantitatively among all possible combinations and represented the panel that achieved the highest sensitivity across all endotypes. The final biomarker panel consisted of C2M, C3M, N-MID, PRO-C2, PRO-C4, and sCTX-I (Table 2).

**Table 1:** Baseline clinical data from the included knee osteoarthritis studies

	IMI-APPROACH (*n* = 226)^a^	CSMC021C2301 (*n* = 947)^b^	MIV-711-201 (*n* = 215)^c^	UBX0101-MUS-201 (*n* = 145)
Sex				
Female	172 (76.1%)	639 (67.5%)	166 (77.2%)	92 (63.4%)
Race				
White	219 (96.9%)	858 (90.6%)	213 (99.1%)	115 (79.3%)
Asian	3 (1.3%)	88 (9.3%)	0 (0.0%)	1 (0.7%)
Black	0 (0.0%)	0 (0.0%)	2 (0.9%)	29 (20.0%)
Other	4 (1.8%)	1 (0.1%)	0 (0.0%)	0 (0.0%)
BMI (kg/m^2^)				
Mean (SD)	27.75 (5.07)	28.86 (4.70)	32.22 (5.62)	29.75 (4.47)
Age (years)				
Mean (SD)	66.50 (7.03)	64.56 (6.67)	61.47 (6.63)	62.88 (9.06)
KL grade				
0	39 (17.8%)	0 (0.0%)	1 (0.5%)	0 (0.0%)
1	60 (27.4%)	0 (0.0%)	50 (23.4%)	24 (16.6%)
2	48 (21.9%)	825 (87.1%)	90 (42.1%)	25 (17.2%)
3	63 (28.8%)	122 (12.9%)	72 (33.6%)	67 (46.2%)
4	9 (4.1%)	0 (0.0%)	1 (0.5%)	29 (20.0%)
JSW medial (mm)				
Mean (SD)	4.28 (1.26)	3.34 (0.95)	-	-
WOMAC pain (%)				
Mean (SD)	29.28 (21.80)	47.53 (15.03)	52.96 (15.46)	53.28 (14.57)
WOMAC function (%)				
Mean (SD)	29.76 (21.45)	47.25 (17.30)	55.72 (15.41)	55.70 (13.87)
WOMAC stiffness (%)				
Mean (SD)	36.94 (25.36)	49.60 (21.80)	55.70 (16.93)	58.88 (17.55)
NRS knee pain (%)				
Mean (SD)	4.53 (2.92)	-	6.14 (1.34)	6.66 (1.41)

The data were based on the target knee information. Higher Western Ontario and McMaster Universities Osteoarthritis Index (WOMAC) and average numeric rating scale (NRS) knee pain scores indicate worse symptoms. JSW, joint space width; KL, Kellgren-Lawrence; *SD,* standard deviation

^a^Missing data: BMI (*n* = 1), KL grade (*n* = 7), medial JSW (*n* = 10), WOMAC pain (*n* = 5), WOMAC function (*n* = 13), WOMAC stiffness (*n* = 2), NRS knee pain (*n* = 2)

^b^Missing data: WOMAC function (*n* = 1)

^c^Missing data: KL grade (*n* = 1)

**Table 2:** Panel of six blood-based biomarkers of tissue turnover with the highest predictive ability of the three molecular endotypes

Biomarker	Biological association	Description
PRO-C2	Cartilage formation	Type IIB N-terminal propeptide of type II collagen [19].
C2M	Cartilage degradation	MMP-8-, -9-, -12-mediated cleavage of type II collagen degradation fragment [20].
sCTX-I	Bone resorption	Cathepsin K-mediated cleavage of β-isomerized type I collagen α1(I) C-terminal telopeptide [21].
N-MID	Bone formation	N-terminal middle fragment of osteocalcin [22].
C3M	Synovial inflammation	MMP-9-mediated degradation of type III collagen [23].
PRO-C4	Inflammation	7 S domain of N-terminal propeptide of type IV collagen [24].

MMP, matrix metalloproteinase

### Endotype-specific treatment effects in former clinical trials

To explore the potential for differential treatment effects among endotype subgroups, biomarker-based endotypes were predicted at baseline in three former interventional trials investigating the efficacy of salmon calcitonin (CSMC021C2301), MIV-711 (MIV-711-201), and UBX0101 (UBX0101-MUS-201) (Table 3). The distribution of predicted endotypes was similar between the placebo and treatment groups within each trial (Supplementary Table 3).

**Table 3:** Knee osteoarthritis interventional trials considered in this study and the endotype hypothesized to exhibit the strongest treatment effect based on the drug mode of action

	CSMC021C2301 (*n* = 947)	MIV-711-201 (*n* = 215)	UBX0101-MUS-201 (*n* = 145)
Participants (pla/tx)	435/512	69/146	32/113
Visits	0, 2 years	0, 26 weeks	0, 12 weeks
Treatment	Salmon calcitonin	MIV-711	UBX0101
*Treatment groups	0.8 mg	100 mg, 200 mg	0.5 mg, 2.0 mg, 4.0 mg
Administration	Oral	Oral	Intra-articular
Primary outcome	JSW, WOMAC pain	NRS knee pain	WOMAC pain
Mode of action	Calcitonin	Cathepsin K inhibitor	p53/MDM2 inhibitor
Drug type	Anti-bone resorptive	Anti-bone resorptive	Senolytic
Endotype match	Structural damage	Structural damage	Inflammatory

*Treatment groups were considered as one group due to small sample sizes. JSW, joint space width; NRS, numeric rating scale; Pla, placebo; Tx, treatment; WOMAC, Western Ontario and McMaster Universities Arthritis

Treatment effects of primary endpoints within endotypes were estimated with linear models, adjusting for age, sex, BMI, KL grade, and baseline levels for each trial, whereas overall differential treatment effects between endotypes were assessed with F tests (Table 4). Overall, the results did not provide strong evidence of differential treatment effects among the predicted endotypes in the trials, although several potential signals were observed. For salmon calcitonin, a significant overall two-year improvement in WOMAC pain was detected when comparing treatment to placebo (-4.38%; 95% CI: -7.17%, -1.59%). Although differences in treatment effects between endotypes were not statistically significant (*p* = 0.486), the structural damage endotype was the only subgroup to show a significant treatment effect versus placebo (-6.19%; 95% CI: -10.55%, -1.83%). The relationship between the SD of the two-year change in WOMAC pain and the probability of belonging to each endotype was explored in the treatment arm of the salmon calcitonin trial (Supplementary Fig. 2). Of note, a 9–16% reduction in outcome variability was observed for the participants in the top 20% quantile probability of belonging to the structural damage endotype (Supplementary Fig. 2). Had this subgroup been enriched in the trial, this reduction in SD could potentially have led to an 18–28% reduction in the sample size needed to demonstrate a statistically significant effect on WOMAC pain (Supplementary Table 4).

For MIV-711, the predicted structural damage endotype showed the strongest numerical 26-week reduction in NRS knee pain compared to placebo. The structural damage endotype had a 26-week NRS knee pain difference of -6.46% (95% CI: -16.23%, 3.31%), low tissue turnover had a change of -5.66% (95% CI: 17.02%, 5.70%), followed by the inflammatory endotype with the smallest numeric difference of -0.09% (-9.78%, 9.60%).

For UBX0101, only the predicted low-tissue turnover endotype showed a numerical 12-week reduction in WOMAC pain compared to placebo, with a difference of -3.91% (95% CI: -17.98%, 10.16%).

**Table 4:** Differences in least-squares mean changes from baseline of primary endpoints between treatment and placebo of predicted knee osteoarthritis endotypes from three trials, and overall assessment of differential treatment effects between endotypes

Primary endpoint	Participants (Pla/Tx)	Mean change from baseline (Pla/Tx)	Treatment effect, LS-mean (95% CI)	*P*-value
Salmon calcitonin (CSMC021C2301)				
Medial JSW (mm)				0.774
Overall	435/512	-0.260/-0.269	-0.009 (-0.095, 0.077)	
Structural damage	180/204	-0.265/-0.250	0.010 (-0.124, 0.145)	
Low tissue turnover	131/157	-0.338/-0.318	0.012 (-0.144, 0.167)	
Inflammatory	124/151	-0.168/-0.245	-0.059 (-0.220, 0.103)	
WOMAC Pain (%)				0.486
Overall	435/512	-20.50/-24.21	-4.38 (-7.17, -1.59)	
Structural damage	180/204	-19.63/-24.99	-6.19 (-10.55, -1.83)	
Low tissue turnover	131/157	-22.66/-23.80	-2.13 (-7.16, 2.90)	
Inflammatory	124/151	-19.46/-23.58	-4.21 (-9.44, 1.01)	
*MIV-711 (MIV-711-201)*				
NRS Knee Pain (%)				0.511
Overall	69/146	-13.04/-16.37	-4.26 (-10.02, 1.50)	
Structural damage	24/57	-10.00/-15.44	-6.46 (-16.23, 3.31)	
Low tissue turnover	17/48	-15.88/-18.96	-5.66 (-17.02, 5.70)	
Inflammatory	28/41	-13.93/-14.63	-0.09 (-9.78, 9.60)	
*UBX0101 (UBX0101-MUS-201)*				
WOMAC Pain (%)				0.951
Overall	32/113	-26.25/-25.27	0.48 (-6.79, 7.75)	
Structural damage	16/45	-28.44/-24.33	2.82 (-7.87, 13.51)	
Low tissue turnover	9/34	-22.22/-26.03	-3.91 (-17.98, 10.16)	
Inflammatory	7/34	-26.43/-25.74	0.72 (-14.21, 15.65)	

Changes in primary endpoints between placebo (pla) and treatment (tx) within the predicted endotypes were estimated using linear models adjusted for age, sex, BMI, Kellgren-Lawrence grade, and baseline levels. Differential treatment effects between endotypes were tested using F-tests with *p*-values shown for the F-tests. Higher Western Ontario and McMaster Universities Osteoarthritis Index (WOMAC) and numeric rating scale (NRS) scores indicate worsening symptoms. CI, confidence interval; JSW, joint space width; LS, least squares

## Discussion

This exploratory, hypothesis-generating study aimed to develop a clinically applicable tool for molecular endotyping based on a minimal panel of biomarkers. The biomarkers included in the prediction model were quantitatively determined based on their ability to predict the three KOA endotypes. This study, for the first time, aimed to evaluate potential differential treatment effects across biomarker-based endotypes using data from prior unsuccessful phase II-III KOA drug trials.

Three endotypes from the IMI-APPROACH consortium were described longitudinally through *k*-means clustering of 19 tissue-turnover biomarkers [8, 9]. To increase the clinical applicability and feasibility of endotyping as a tool for clinical trial recruitment, a smaller panel of biomarkers was deemed necessary [12]. To balance the applicability and accuracy of molecular endotyping, this study explored the possibility of predicting the three endotypes using a multinomial logistic regression model based on a minimal panel of six blood-based biomarkers. The prediction model was trained on IMI-APPROACH and the final model, consisting of C2M, C3M, N-MID, PRO-C2, PRO-C4, sCTX-I, along with age, sex, and BMI, achieved a sensitivity of 90% in the test set during model training. For the selection of the optimal panel of six biomarkers as input parameters to the prediction model, it was decided to select the panel that achieved the highest average sensitivity across all three endotypes. This was done as the model was intended as a trial enrichment tool where a certain level of false negatives can be acceptable and a level of biological overlap between the endotypes may exist. However, increased sensitivity inevitably comes as a trade-off of specificity. To overcome the reduction in specificity, it is possible to adjust the threshold of endotype membership degrees obtained from the prediction model. This was exemplified in the investigation of the relationship between the SD of the primary outcome for CSMC021C2301 and increasing thresholds of predicted endotype membership probabilities.

While it was possible to predict endotypes using only six biomarkers, reducing the number of disease-relevant parameters in the model comes at the cost of accuracy. Although 19 biomarkers have been used to describe the three endotypes longitudinally [9], even utilizing a reduced set of six biomarkers for OA drug trial recruitment strategies will prove challenging from a regulatory perspective and remains exploratory in its current state [25, 26]. However, to encapsulate the vast heterogeneity and complexity of OA pathology, it may become necessary to move beyond testing only single biomarkers. It may be beneficial to develop strategies for OA drug development that embrace smaller panels of disease-relevant and/or pharmacodynamic biomarkers, whether biochemical or imaging.

**Fig. 1:**
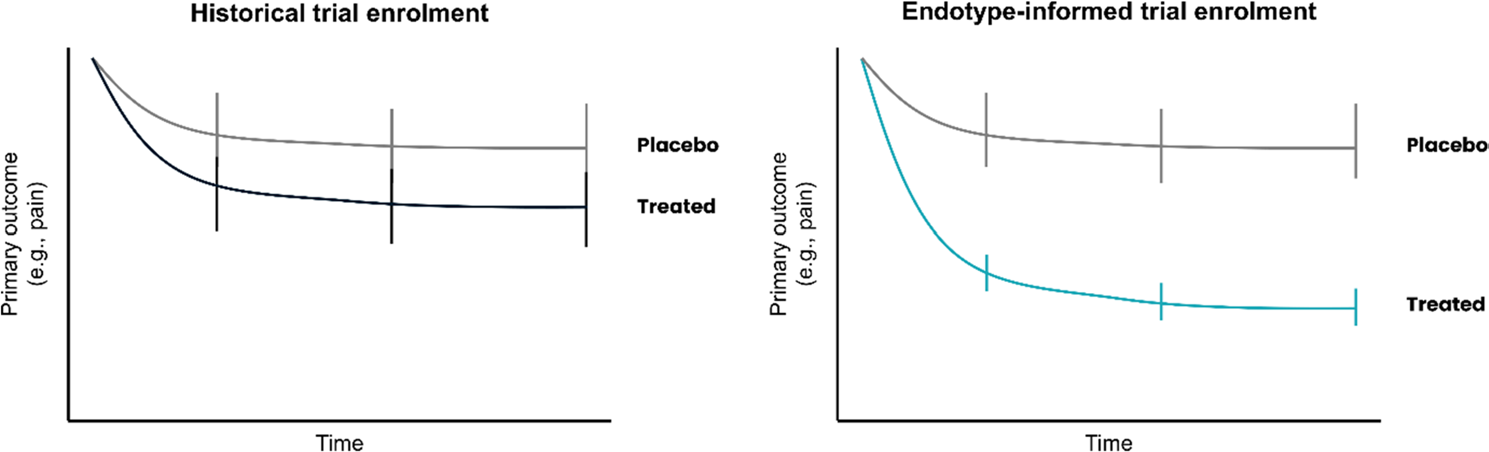
Proposed benefits of endotype-informed osteoarthritis drug trials. Enriching for the right endotype subgroups may potentially increase the effect size and reduce outcome variability to maximize the likelihood of demonstrating clinically meaningful treatment effect

Overall, the results of this study did not demonstrate statistically significant differential treatment effects between the predicted endotypes. As the overall analysis did not reach statistical significance and this study performed post hoc analysis of trial data not powered for subgroup analysis, drawing statistical inferences from differences between the endotype subgroups is not appropriate [27, 28]. As such, the novel application of endotype-informed subgroup analysis in historical OA trial data should be interpreted as hypothesis-generating and of exploratory nature. As a very limited number of trials with proven effect and available data exist in OA, the potential for endotype-specific treatment effects was explored in historical trial data that failed to meet their primary endpoints. By assessing the potential for differential treatment effects between the endotypes in this study, we assumed that at least a subset of the study participants had target engagement or even a true response to treatment. However, it cannot be ruled out that the treatments simply did not work. This limited the power to detect interactions between endotypes and treatments, and we appreciate and emphasize that we explored patterns and trends as a proof-of-concept of endotyping in an OA trial context. One way to support these assumptions would be to assess the pharmacokinetics of each participant. Unfortunately, such data were not available, which is a significant limitation of this study. However, some interesting observations were made. For treatment with MIV-711, the structural damage endotype showed the largest numerical 26-week reduction in NRS knee pain compared with placebo. For treatment with salmon calcitonin, only the structural damage endotype showed a statistically significant two-year reduction in WOMAC pain compared with placebo, yielding a modestly larger effect size than the overall study population. When investigating the relationship between outcome variability and the probability of belonging to each endotype in the salmon calcitonin trial, we found that the participants in the top 20% strongest probability of belonging to the structural damage endotype had a 9–16% reduction in the SD of the two-year change in WOMAC pain. Had this subgroup been enriched in the trial, this reduction in SD could potentially have led to an 18–28% reduction in the number of participants needed to be enrolled. These findings may suggest that aligning treatments with specific endotype subgroups, such as targeting anti-bone resorptive treatments to OA subpopulations with bone- and cartilage-damage-driven endotypes, may be beneficial and merits further investigation. In recent years, other disease areas have successfully implemented endotype-informed clinical trials to improve trial outcomes, such as in patients with angina and unobstructed coronary arteries [29] as well as difficult-to-treat asthma [30]. Likewise, we propose endotype-informed clinical trials may potentially both increase the effect size and reduce the outcome heterogeneity to maximize the likelihood of demonstrating clinically meaningful treatment effects (Fig. 1).

The limitations of this study include the small sample size. For MIV-711 and UBX0101 which assessed multiple drug doses, all treatment groups were pooled to overcome very small study sizes when stratified into the three endotypes. A clearer separation of treatment effects between the endotypes may have been observed if only the largest treatment dose groups were considered in the analysis. Differences in disease duration may also have influenced both the clinical characteristics and response to treatment of the trial participants. As this information was not available for all of the trials, the results of this study were limited by the inability to account for disease duration. Another limitation of this study was the need for imputation of missing data, especially for biomarkers needed for the prediction model in treatment studies. For the UBX0101-MUS-201 trial, the biomarkers sCTX-I and N-MID had 27% missing data owing to insufficient sample volume. The extensive imputation required for two of the six biomarkers used in the prediction model may have influenced the reliability of the endotype predictions.

The endotype prediction model was based on a multinomial logistic regression model. It is conceivable that the endotypes may overlap and that the biomarkers included in the model are not biologically decoupled [3, 9]. Therefore, providing discrete endotype assignments may lead to misclassification of some participants that exhibit molecular properties of belonging to more than one endotype, and is a limitation of the prediction model.

While a senolytic agent such as UBX0101 may be associated with anti-inflammatory properties, a major limitation of this study was the lack of a trial assessing the efficacy of a true anti-inflammatory treatment such as an anti-IL-1 agent. A well-suited study could have been the Canakinumab Anti-Inflammatory Thrombosis Outcomes Study (CANTOS) in which treatment with the IL-1β inhibitor was found to lower the incidence rate of total joint replacements by 40–47% in a secondary analysis of OA participants with a previous history of myocardial infarction and elevated levels of hsCRP [31]. In such cases, a potentially larger treatment effect may have been achieved in the inflammatory endotype subgroup. Another limitation of the prediction model is that it was trained and tested in a predominantly White European population. Therefore, the generalizability of the model to populations with different demographic characteristics is unknown and warrants further study. The endotyping model was also limited to KOA; therefore, its applicability to other OA types, such as hand OA, is also unknown. Finally, the biomarkers used to predict endotypes reflect specific pathobiological processes. Consequently, the endotype prediction model may not be suitable for some drug trials in which the mode of action does not align with the biomarkers. Other markers, such as imaging biomarkers, may therefore be more appropriate for enrichment based on specific treatment. Examples include the use of magnetic resonance imaging (MRI)-based biomarkers, such as cartilage thickness or cartilage composition biomarkers, when matched with cartilage anabolic treatments [32] as well as MRI Osteoarthritis Knee Score (MOAKS)-based effusion-synovitis when matched with anti-inflammatory treatments.

## Conclusions

In conclusion, this exploratory study highlights the potential to predict KOA endotypes using a minimal panel of tissue-turnover biomarkers, potentially providing an applicable tool to improve the recruitment of appropriate OA subpopulations for clinical trials. To our knowledge, this is the first study to assess endotype-specific treatment effects in previous phase II-III KOA drug trials. Although no overall differential treatment effects were observed between the predicted endotypes, the nominal treatment effects of anti-bone resorptive treatments and reduced outcome variability in the structural damage endotype may subtly suggest that aligning treatments with molecular endotypes according to the drug mode of action may be beneficial. However, this study remains hypothesis-generating in nature and further investigations are needed. Prospective and adequately powered trials that incorporate stratified randomization or enrichment of biomarker-based endotypes will be key to establishing their true clinical utility. In doing so, this may advance the acceptance and implementation of endotype-guided OA drug development.

## Supplementary Information

Supplementary Material 1.

## Data Availability

Data from the IMI-APPROACH can be provided upon reasonable request to the IMI-APPROACH steering committee.

## Abbreviations

BMI: Body mass index
CI: Confidence interval
COMP: Cartilage oligomeric matrix protein
HA: Hyaluronic acid
hsCRP: High-sensitivity C-reactive protein
IMI-APPROACH: Innovative Medicines Initiative Applied Public-Private Research enabling Osteoarthritis Clinical Headway
IQR: Interquartile range
JSW: Joint space width
KL: Kellgren-Lawrence
KOA: Knee osteoarthritis
LMM: Linear mixed model
LS: Least-squares
N-MID: N-terminal middle fragment of osteocalcin
NRS: Numeric rating scale
OA: Osteoarthritis
OAI: Osteoarthritis Initiative
SD: Standard deviation
VICM: Citrullinated vimentin
WOMAC: Western Ontario and McMaster Universities Osteoarthritis Index
