# Supplementary material for: Therapeutic subtypes of knee osteoarthritis: differential treatment effects among predicted endotypes in past clinical trials
Source: Arthritis Res Ther. 2026 May 7;28:133. doi: 10.1186/s13075-026-03825-7 (PMC13317274; doi:10.1186/s13075-026-03825-7)
Supplement: Supplementary file 1 — Supplementary Material 1. [file 13075_2026_3825_MOESM1_ESM.zip › Supplementary file.docx]

**Supplementary tables**

**Supplementary Table 1.** Number of imputed biomarker data points across the four knee osteoarthritis studies included in this work.

|  | **IMI-APPROACH** | **CSMC021C2301 (placebo)** | **CSMC021C2301 (treatment)** | **MIV-711-201** | **UBX0101-MUS-201** |
| --- | --- | --- | --- | --- | --- |
| **Visit(s)** | Month 6-24 | Baseline | Baseline | Baseline | Baseline |
| **Data points** | 678 | 435 | 512 | 215 | 145 |
| **ARG** | - | 2 (0.46%) | - | - | - |
| **C1M** | 4 (0.52%) | 0 (0.00%) | - | - | - |
| **C2M** | 18 (2.33%) | 3 (0.69%) | 0 (0.00%) | 0 (0.00%) | 0 (0.00%) |
| **C3M** | 8 (1.04%) | 0 (0.00%) | 0 (0.00%) | 0 (0.00%) | 0 (0.00%) |
| **C10C** | 1 (0.13%) | - | - | - | - |
| **COMP** | 1 (0.13%) | - | - | - | - |
| **CRPM** | 1 (0.13%) | 0 (0.00%) | - | - | - |
| **HA** | 1 (0.13%) | - | - | - | - |
| **hsCRP** | 0 (0.00%) | - | - | - | - |
| **N-MID** | 0 (0.00%) | 0 (0.00%) | 0 (0.00%) | 2 (0.93%) | 39 (26.90%) |
| **PRO-C1** | 6 (0.78%) | - | - | - | - |
| **PRO-C2** | 15 (1.94%) | 0 (0.00%) | 0 (0.00%) | 0 (0.00%) | 0 (0.00%) |
| **PRO-C4** | 4 (0.52%) | 45 (10.34%) | 19 (3.71%) | 0 (0.00%) | 3 (2.07%) |
| **sCTX-I** | 5 (0.65%) | 0 (0.00%) | 0 (0.00%) | 0 (0.00%) | 39 (26.90%) |
| **uCTX-I** | - | 26 (5.98%) | - | - | - |
| **uCTX-II** | - | 26 (5.98%) | - | - | - |
| **VICM** | 4 (0.52%) | 0 (0.00%) | - | - | - |
| **Total** | 68 (0.63%) | 102 (1.95%) | 19 (0.62%) | 2 (0.16%) | 81 (9.31%) |

Missing data were due to insufficient sample volume. The biomarkers C2M, C3M, N-MID, PRO-C2, PRO-C4, and sCTX-I were included in the final prediction models.

**Supplementary Table 2.** Average performance of the top 10 endotype prediction models based on panels of six blood-based biomarkers of tissue turnover, age, sex, and BMI.

|  | **Sensitivity** | | | | **Specificity** | | | |
| --- | --- | --- | --- | --- | --- | --- | --- | --- |
| **Biomarker panel** | **All** | **IF** | **LTT** | **SD** | **All** | **IF** | **LTT** | **SD** |
| **C2M, C3M, N-MID, PRO-C2, PRO-C4, sCTX-I** | 0.896 | 0.920 | 0.892 | 0.877 | 0.945 | 0.956 | 0.939 | 0.940 |
| C2M, C3M, PRO-C1, PRO-C2, PRO-C4, sCTX-I | 0.890 | 0.880 | 0.874 | 0.916 | 0.943 | 0.964 | 0.961 | 0.904 |
| C2M, PRO-C1, PRO-C2, PRO-C4, sCTX-I, VICM | 0.888 | 0.887 | 0.860 | 0.917 | 0.942 | 0.967 | 0.964 | 0.894 |
| C1M, C2M, PRO-C1, PRO-C4, sCTX-I, VICM | 0.884 | 0.900 | 0.849 | 0.904 | 0.941 | 0.971 | 0.943 | 0.909 |
| C2M, C3M, C10C, PRO-C1, PRO-C4, N-MID | 0.884 | 0.894 | 0.896 | 0.860 | 0.940 | 0.964 | 0.907 | 0.950 |
| C2M, C3M, PRO-C1, PRO-C2, PRO-C4, N-MID | 0.883 | 0.880 | 0.893 | 0.877 | 0.938 | 0.959 | 0.936 | 0.919 |
| C2M, C10C, PRO-C1, PRO-C4, N-MID, VICM | 0.883 | 0.903 | 0.861 | 0.883 | 0.940 | 0.966 | 0.930 | 0.922 |
| C2M, PRO-C1, PRO-C2, PRO-C4, N-MID, VICM | 0.879 | 0.910 | 0.853 | 0.876 | 0.936 | 0.962 | 0.936 | 0.912 |
| PRO-C2, C3M, C2M, COMP, PRO-C4, N-MID | 0.879 | 0.903 | 0.839 | 0.894 | 0.936 | 0.967 | 0.951 | 0.889 |
| sCTX-I, CRPM, PRO-C2, C3M, PRO-C1, PRO-C4 | 0.876 | 0.851 | 0.869 | 0.908 | 0.935 | 0.962 | 0.960 | 0.882 |

The endotype prediction models were based on a multinomial logistic regression model. The models were trained and tested on participants with knee osteoarthritis from IMI-APPROACH.[9,17] IF, inflammatory endotype; LTT, low tissue turnover endotype; SD, structural damage endotype.

**Supplementary Table 3**. Distribution of predicted endotypes across interventional knee osteoarthritis trials included in this study.

|  | **CSMC021C2301** | | **MIV-711-201** | | **UBX0101-MUS-201** | |
| --- | --- | --- | --- | --- | --- | --- |
| **Predicted endotype** | **Pla** | **Tx** | **Pla** | **Tx** | **Pla** | **Tx** |
| Structural damage | 180 (41%) | 204 (40%) | 24 (35%) | 57 (39%) | 16 (50%) | 45 (40%) |
| Low turnover | 131 (30%) | 157 (31%) | 17 (25%) | 48 (33%) | 9 (28%) | 34 (30%) |
| Inflammatory | 124 (29%) | 151 (29%) | 28 (41%) | 41 (28%) | 7 (22%) | 34 (30%) |
| Total | 435 | 512 | 69 | 146 | 32 | 113 |

Pla, placebo; Tx, treatment.

**Supplementary Table 4.** Change in standard deviation (SD) of two-year change in WOMAC pain in treatment arm of the oral salmon calcitonin trial [13] for every decile group of the probability of membership to the structural damage endotype, and the corresponding sample size needed in each treatment arm to demonstrate statistically significant effect on WOMAC pain.

|  | Structural damage endotype probability decile cutoffs | | | | | | | | | |
| --- | --- | --- | --- | --- | --- | --- | --- | --- | --- | --- |
|  | 0% | 10% | 20% | 30% | 40% | 50% | 60% | 70% | 80% | 90% |
| ∆SD (%) | - | 0.5 | 0.1 | -2.6 | -2.2 | -3.6 | -1.5 | -3.9 | -9.3 | -15.6 |
| Sample size needed | 306 | 310 | 307 | 291 | 293 | 285 | 297 | 283 | 252 | 219 |
| ∆Sample size needed (%) | - | 1.3 | 0.3 | -4.9 | -4.2 | -6.9 | -2.9 | -7.5 | -17.6 | -28.4 |

The sample size needed in each treatment arm to demonstrate a statistically significant difference of 32/500 mm in Western Ontario and McMaster Universities Osteoarthritis Index (WOMAC) pain was calculated using a power of 96% [13] and standard deviation of each endotype probability decile group.


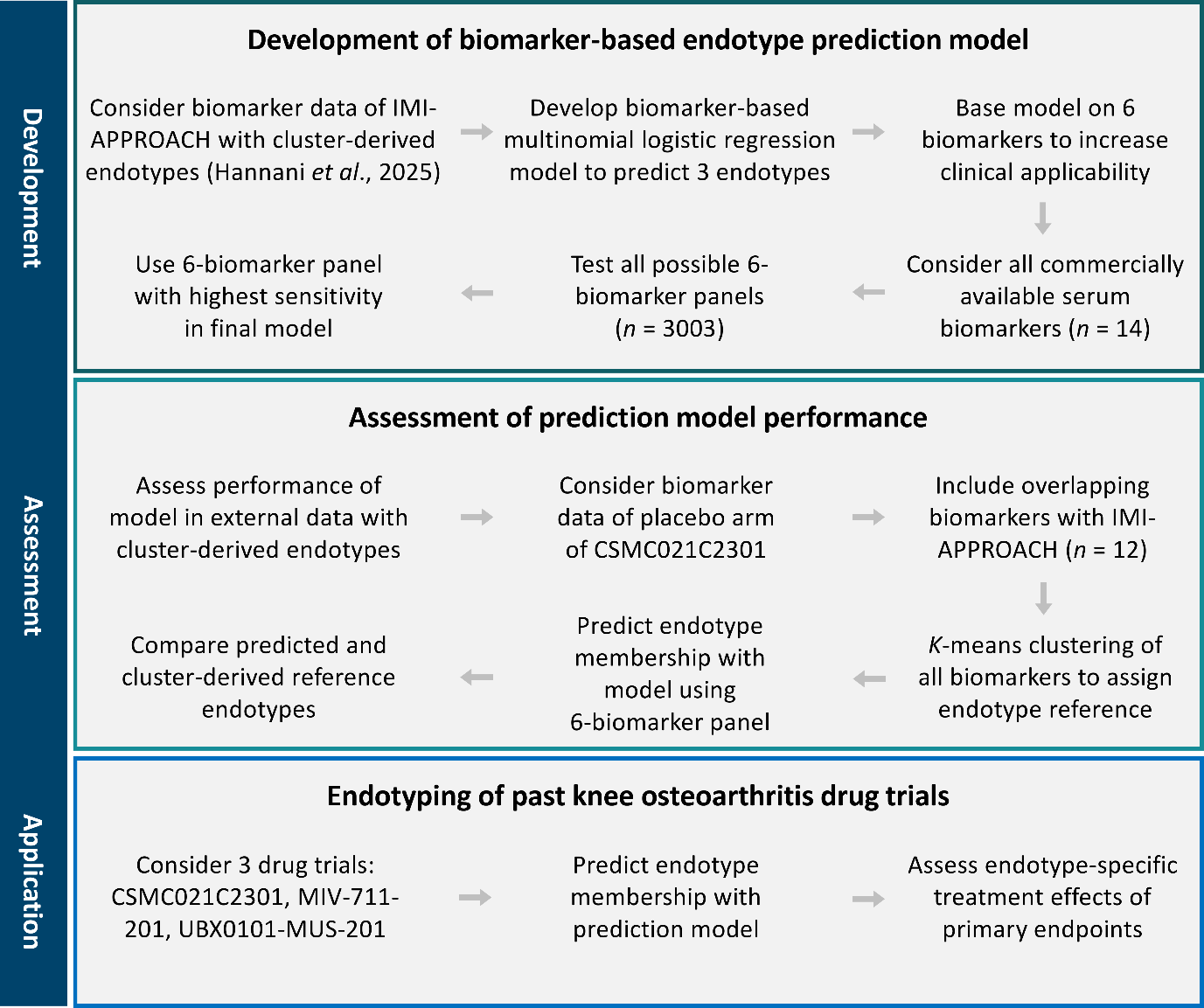


**Supplementary Figure 1**. Analytical framework for the development, assessment, and application of biomarker-based endotype prediction model.


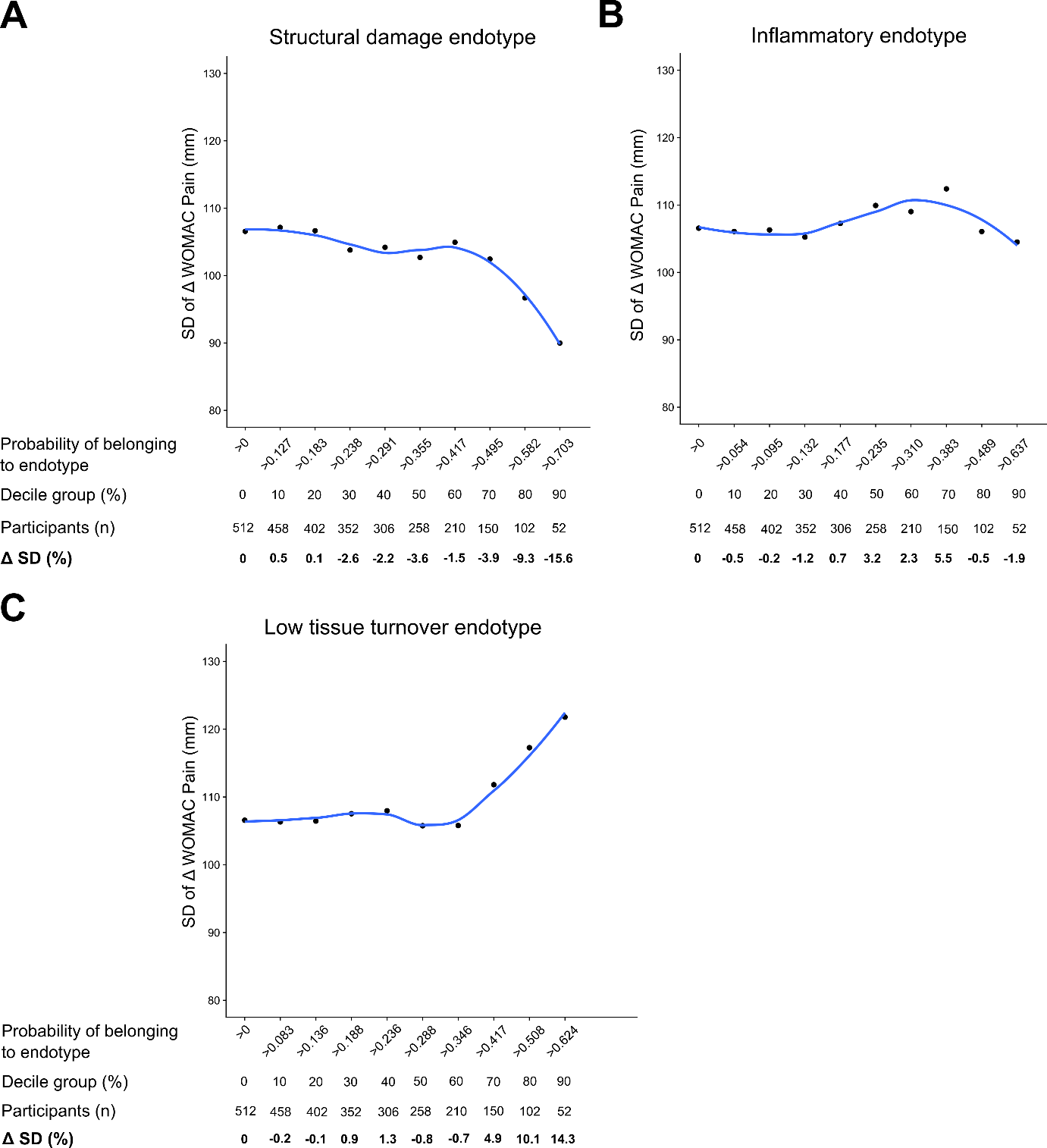


**Supplementary Figure 2.** Relationship between outcome variability of WOMAC pain and the probability of belonging to each endotype in the treatment arm of the salmon calcitonin trial (CSMC021C2301). The standard deviation (SD) of the two-year change in WOMAC pain was computed for every decile cutoff of the endotype probabilities obtained from the multinomial endotype prediction model. WOMAC, Western Ontario and McMaster Universities Osteoarthritis Index.
