# Supplementary material for: Therapeutic subtypes of knee osteoarthritis: differential treatment effects among predicted endotypes in past clinical trials
Source: Arthritis Res Ther. 2026 May 7;28:133. doi: 10.1186/s13075-026-03825-7 (PMC13317274; doi:10.1186/s13075-026-03825-7)
Supplement: Supplementary file 1 — Supplementary Material 1. [file 13075_2026_3825_MOESM1_ESM.zip › Supplementary Material.docx]

**Supplementary Material**

**Assessment of performance of endotype prediction model**

It has previously been shown that the three endotypes originally described in the IMI-APPROACH could be recaptured in the OAI study by clustering a smaller subset of overlapping biomarkers [1]. To explore the accuracy of predicting the endotypes with a reduced set of biomarkers, the performance of the prediction model was assessed using baseline data from the placebo arm of the oral salmon calcitonin trial (CSMC021C2301) [2]. Longitudinal data were included from participants who completed the study and had measurements for more than five biomarkers at both baseline and month 24, resulting in 435 participants in the placebo arm.

As proof of concept, the placebo arm of CSMC021C2301 was clustered based on all available serum (s) and urine (u) biomarker data (*n* = 12) that overlapped with Hannani *et al*. (2025) to replicate the three endotypes described in the IMI-APPROACH [1,3]. The biomarkers ARG, C1M, C2M, C3M, CRPM, N-MID, PRO-C2, PRO-C4, sCTX-I, uCTX-I, uCTX-II, and VICM were included [3,4]. The biomarker values were pre-processed as described above. As performed by Hannani *et al*. (2025), *k*-means clustering (*k* = 3) was applied to assign participants to the structural damage, inflammatory, and low tissue turnover endotypes [3]. The performance of the endotype prediction model was evaluated by the concordance between predicted and cluster-derived reference endotype labels.

Overall, the endotype prediction model achieved an accuracy of 73% (95% confidence interval [CI]: 69%, 77%). The highest sensitivity was found for the structural damage endotype (85%), followed by the inflammatory endotype (71%), and low tissue turnover endotype (63%) (Tables 1-2).

**References**

1. Angelini F, Widera P, Mobasheri A, Blair J, Struglics A, Uebelhoer M, et al. Osteoarthritis endotype discovery via clustering of biochemical marker data. Ann Rheum Dis. 2022 May;81(5):666–75.

2. Karsdal MA, Byrjalsen I, Alexandersen P, Bihlet A, Andersen JR, Riis BJ, et al. Treatment of symptomatic knee osteoarthritis with oral salmon calcitonin : results from two phase 3 trials. Osteoarthr Cartil. 2015;23(4):532–43.

3. Hannani MT, Thudium CS, Gellhorn AC, Larkin J, Karsdal MA, Lisowska-Petersen Z, et al. Longitudinal stability of molecular endotypes of knee osteoarthritis patients. Osteoarthr Cartil. 2025;33(1):166–75.

4. Hannani MT, Thudium CS, Karsdal MA, Mobasheri A, Uebelhoer M, Larkin J, et al. From biochemical markers to molecular endotypes of osteoarthritis: a review on validated biomarkers. Expert Rev Mol Diagn. 2024;24(1–2):23–38.

**Figures**


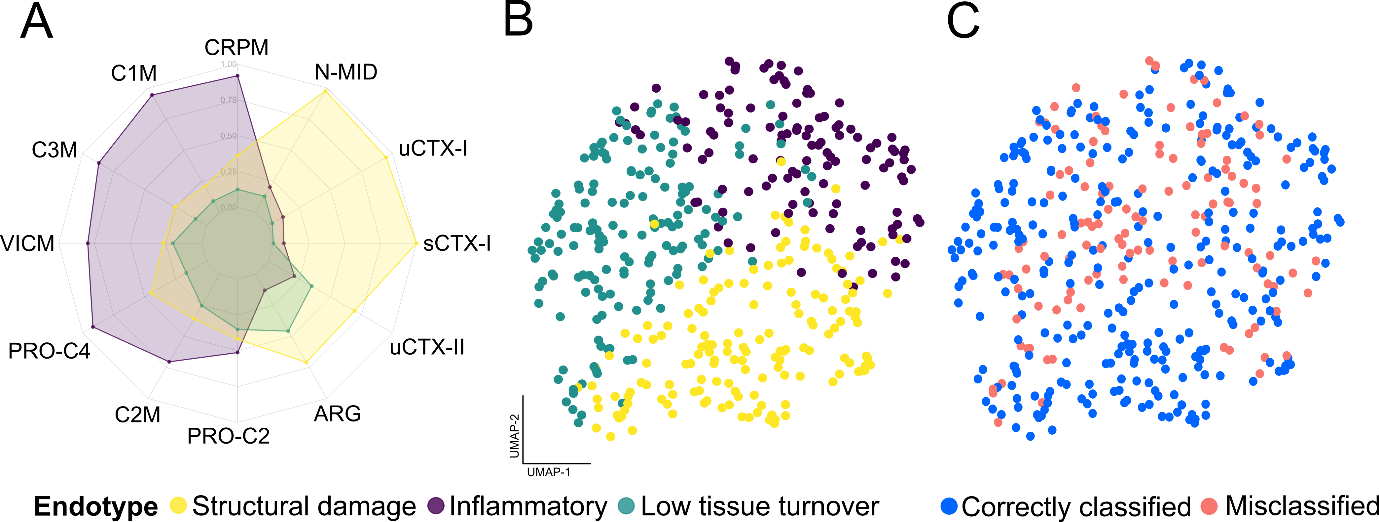


**Figure 1.** Proof-of-concept endotyping of the placebo arm (*n* = 435) of the salmon calcitonin trial (CSMC021C2301) [2]. **A)** Biomarker profile of the three endotypes, showing min-max normalized values of sex-specific z-score scaled concentrations. Uniform Manifold Approximation and Projections (UMAPs) of **B)** cluster-derived reference endotypes, and **C)** endotype classifications from the endotype prediction model.

**Tables**

**Table 1.** Confusion matrix of the endotype multinomial logistic regression model performance in the placebo arm (*n* = 435) of the salmon calcitonin trial (CSMC021C2301) [2].

|  | **Cluster-derived endotype** | | |
| --- | --- | --- | --- |
| **Predicted endotype** | Inflammatory | Low tissue turnover | Structural damage |
| Inflammatory | 85 | 20 | 19 |
| Low tissue turnover | 25 | 102 | 4 |
| Structural damage | 9 | 40 | 131 |

**Table 2.** Endotype-specific performance of the endotype multinomial logistic regression prediction model in the placebo arm (*n* = 435) of the salmon calcitonin trial (CSMC021C2301) [2].

|  | Inflammatory | Low tissue turnover | Structural damage |
| --- | --- | --- | --- |
| Sensitivity | 0.7143 | 0.6296 | 0.8506 |
| Specificity | 0.8766 | 0.8938 | 0.8256 |
